# Supplementary material for: Assessing pain severity and treatment outcomes in patients with low back pain: A Structural equation modeling approach at the center for the rehabilitation of the Paralysed, Bangladesh
Source: PLoS One. 2024 May 31;19(5):e0303939. doi: 10.1371/journal.pone.0303939 (PMC11142540; doi:10.1371/journal.pone.0303939)
Supplement: S1 File — (ZIP) [file pone.0303939.s001.zip › ethics statement.pdf]

CRP
Centre for the Rehabilitation of the Paralysed
পক্ষাঘাতগ্রস্তদের পুনর্বাসন কেন্দ্র (সিআরপি)
Centre for the Rehabilitation of the Paralysed (CRP)
a project of the Trust for the Rehabilitaton of the Paralysed
Head Office: CRP- Savar, CRP- Chapain, Savar Dhaka-1343, Bangladesh
Tel: +880 02 7745464-5, Fax: 7745069, E-mail: contact@crp-bangladesh.org, www. crp-bangladesh.org

Ref: *Date:*

CRP-R&E-0401-239 26.01.2021

To

Mohammad Arifur Rahman

Ref: Study Title "A Structural Equation Modeling to Determine the Pain Severity and Treatment of the Patients with Low Back Pain Attending at Center for the Rehabilitation of the Paralysed, Bangladesh".

Sub: Approval of documents for Study Title "A Structural Equation Modeling to Determine the Pain Severity and Treatment of the Patients with Low Back Pain Attending at Center for the Rehabilitation of the Paralysed, Bangladesh".

Dear Concerns,

The CRP Ethics Committee reviewed and discussed your application to conduct the research entitled ""A Structural Equation Modeling to Determine the Pain Severity and Treatment of the Patients with Low Back Pain Attending at Center for the Rehabilitation of the Paralysed, Bangladesh".

The following documents were reviewed:

SL. No	Documents	**Version**	*Dated*	*Copy*
	Protocol	-	19.01.2021	1

The following members of the ethics committee reviewed the protocol on 19 January 2021

**SL. No**	**Name**	**Role in EC**	**Affiliation with Institute (Yes/No) If yes, Specify.....**
1.	Prof. Dr. Mohammad Alamgir Kabir	Chair of CRP-EC	No
2.	Rafiul Karim	Member Secretary	Yes, Research, Monitoring & Evaluation Officer
3.	Nasirul Islam	Executive Member	Yes, Principal (Acting), BHPI
4.	Julker Nayan	Executive Member	Yes, Head of Occupational Therapy Dept.
5.	Mohammad Anwar Hossain	Executive Member	Yes, Head of Physiotherapy Dept.

পক্ষাঘাতগ্রস্তদের পুনর্বাসন কেন্দ্র (সিআরপি)
CRP
Centre for the Rehabilitation of the Paralysed
Centre for the Rehabilitation of the Paralysed (CRP)
a project of the Trust for the Rehabilitaton of the Paralysed
Head Office: CRP- Savar, CRP- Chapain, Savar Dhaka-1343, Bangladesh
Tel: +880 02 7745464-5, Fax: 7745069, E-mail: contact@crp-bangladesh.org, www. crp-bangladesh.org

Ref: Date:

6.	Sharmin Hasnat		Yes, Sr. SLT & Acting Head and Lecturer, Spech and Language Therapy Department.
7.	Md. Obaidur Rahman		No
8.	Md. Mizanur Rahman		Yes, Lecturer

We confirm that neither you nor your study team members participated in the deliberations of the Ethics Committee & did not vote on the proposal for this study.

We approve the research to be conducted in its presented form at Centre for Rehabilitation of the Paralysed Ethics Committee (CRP-EC)

The CRP Ethics Committee expects to be informed about the progress of the study, andy SAE occurring in the course of the study, any changes in the protocol and patient information/informed consent and asks to be provided a copy of the final report.

Please submit to the EC the status report of the study as per EC SOP's

The EC is organized & operates according to the requirements of Declaration of Helsinki and ICH-GCP, local regulatory requirements and guidelines

Your Sincerely

Rafiul Karim

Research, Monitoring & Evaluation Officer, CRP.
