# Supplementary material for: Assessing pain severity and treatment outcomes in patients with low back pain: A Structural equation modeling approach at the center for the rehabilitation of the Paralysed, Bangladesh
Source: PLoS One. 2024 May 31;19(5):e0303939. doi: 10.1371/journal.pone.0303939 (PMC11142540; doi:10.1371/journal.pone.0303939)
Supplement: S1 File — (ZIP) [file pone.0303939.s001.zip › low back pain question.pdf]

Characteristics and its associated physiotherapy management for the patient with low back pain at CRP in Bangladesh

Part: 1- Personal details:

1.1 Patients name:

1.2 Age:

1.3Sex:

1. Male	2. Female

1.4 Height:

1.5 Weight:

1.6 Address:

Village:	Post office:	Thana:	District:

1.7 Referring physician name:

Part: 2-Socio-demographic information

2.1 Occupation:

1. Farmer	2. Day labor	3.Serviceholder	4. Garments worker
5. Driver	6.Rikshawola	7.Businessman	8.Unemployment
9. Housewife	10.Teacher	11.Student	12.Others

2.2 Marital status:

1. Married	2. Unmarried	3.Widow	4. Divorce

2.3 Family size:

1. Small family	2. Large family

2.4 Number of Children:

2.5 Living place:

1. Urban	2. Rural

2.6 Educational status:

1.Illiterate	2.Primary	3.Secondary	4. HSC passed	5. Graduate & Masters

2.7 Religion:

1. Islam	2. Hindu	3. Christian	4.Buddhist

2.8 Smoking:

1. Yes	2. No

2.9 How many days of work have you missed because of pain during the past 18 months?

a) 0 days	b) 1-2 days	c) 3-7 days	d) 8-14 days	e) 15-30 days
f) 1 month	g) 2 months	h) 3-6 months	i) 6-12 months	j) over a year
k) Not a single day				

2.10 How long have you been suffering from the current pain problem?

a) 0-1 month	b) 1-2 months	c) 3-4 months	d) 4-5 months	e) 6-8 months	f) 9-11 months

Initial & Discharge questions

Part: 3- Dallas Pain questionnaire

3.1 How bad do you suffer because of the pain?

a) No pain
b) Slight pain
c) Moderate pain
d) Severe pain

3.2 How much do you suffer because of the pain at night?

a) No pain
b) Slight pain
c) Moderate pain
d) Severe pain

3.3 Does the pain interfere your life?

a) No problem
b) Slightly problem
c) Moderately problem
d) Severely problem
e) Total change in life style

3.4 How much pain do you feel during your daily life activities?

a) No pain
b) Slight pain
c) Moderate pain
d) Severe pain

3.5 How stiff is your back pain?

a) No stiffness
b) Slightly stiffness
c) Moderately stiffness
d) Severely stiffness
e) Worse possible stiffness

3.6 Does the pain interfere your walking?

a) No problem
b) Slightly problem
c) Moderately problem
d) Severely problem
e) Cannot walk at all

3.7 Does the pain prevent you from standing still?

a) Can stand as long as I want
b) Can stand few times
c) Can stand long time
e) Cannot stand at all

3.8 Does the pain prevent you from twisting?

a) No twisting at all
b) Slightly twisting
c) Moderately twisting
d) Severely twisting

3.9 Does the pain allow you to sit in an upright hard chair?

a) Can sit as long as I want
b) Can sit few times as long as I want
c) Can sit long time as long as I want
d) Cannot use a hard chair at all

3.10 Does the pain allow you to sit in a soft arm chair?

a) Can sit as long as I want
b) Can sit few times as long as I want
c) Can sit long time as long as I want
d) Cannot use a soft arm chair at all

3.11 Do you have low back pain when lying in a bed?

a) No pain
b) Slight pain
c) Moderate pain
d) Severe pain

3.12 Does your pain interfere with your work?

a) No problem
b) Slightly problem
c) Moderately problem
d) Severely problem
e) Cannot work at all

3.13 Did you have to change your jobs because of back pain?

a) No change
b) Change

<u>Part: 4- Low back pain Disability Index</u>

4.1: Pain Intensity

1. I have no pain at the moment
2. The pain is slight at the moment
3. The pain is moderate at the moment
4. The pain is severe at the moment
5. The pain is the worst imaginable at the moment

4.2: Personal Care

1. I can look after myself normally without causing extra pain
2. I can look after myself normally but it causes extra pain
3. It is painful to look after myself and I am slow and careful
4. I need some help but can manage most of my personal care
5. I need help every day in most aspects of self-care
6. I do not get dressed, I wash with difficulty and stay in bed

4.3: Lifting

1. I can lift heavy weights without extra pain
2. I can lift heavy weights but it gives extra pain
3. I can only lift very light weights
4. I cannot lift or carry anything

4.4: Reading

1. I can read as much as I want to with no pain in my back
2. I can read as much as I want to with slight pain in my back
3. I can read as much as I want with moderate pain in my back
4. I can hardly read at all because of severe pain in my back
5. I cannot read at all

4.5: Headaches

1. I have no headaches at all
2. I have slight headaches, which come infrequently
3. I have moderate headaches, which come infrequently
4. I have severe headaches, which come frequently
5. I have headaches almost all the time

4.6: Concentration

1) I can concentrate fully when I want to with no difficulty

2) I can concentrate fully when I want to with slight difficulty
3) I can concentrate fully when I want to with moderate difficulty
4) I can concentrate fully when I want to with severe difficulty
5) I cannot concentrate at all

4.7: Work

1. I can do as much work as I want to
2. I can only do my usual work, but no more
3. I cannot do my usual work
4. I can hardly do any work at all
5. I can't do any work at all

4.8: Sleeping

1. I have no trouble sleeping
2. My sleep is slightly disturbed (less than 1 hr sleepless)
3. My sleep is mildly disturbed (1-2 hrs sleepless)
4. My sleep is moderately disturbed (2-3 hrs sleepless)
5. My sleep is greatly disturbed (3-5 hrs sleepless)
6. My sleep is completely disturbed (5-7 hrs sleepless)

4.9: Recreation

1. I am able to engage in all my recreation activities with no back pain at all
2. I am able to engage in all my recreation activities, with some pain in my back
3. I can hardly do any recreation activities because of pain in my back
4. I can't do any recreation activities at all

Part: 5-Treatment Oriented questions

5.1: Are you taking the physiotherapy treatment regularly (According to dose?)

a) Yes

b) No

If "No" answer the question no 2

5.2: Why are you taking irregular treatment?

a) Personal problem

b) Schedule problem

5.3: Is your patient taking the physiotherapy treatment regularly (According to schedule?)

a) Yes

b) No

5.4: Estimated number of treatment session for first time injury. …………………………..?

5.5: Estimated number of treatment session for 2nd time injury (1st time incomplete dose………………………...?

5.6: Have your patient any other problem which impacting on your physiotherapy Treatment? (Pathological disease or Operation)

a) Yes
b) No

Day-1

a) General pain intensity ……………………………?

b) Treatment protocol (Specific/Need based)

I) Name:

a)

b)

c)

d)

II) Structure (Injured ⇔ Rx):

a)

b)

c)

d)

III) Dose:

a)

b)

c)

d)

IV) Outcome

I) Patient's perception: ____

II) Physiotherapist's perception: ____

Day-2

a) General pain intensity……………………...?

b) Treatment protocol (Specific/Need based)

I) Name:

a)

b)

c)

d)

II) Structure (Injured ⇔ Rx):

a)

b)

c)

d)

III) Dose:

a)

b)

c)

d)

IV) Outcome

I) Patient’s perception: ____

II) Physiotherapist’s perception: ____

Day-3

a) General pain intensity ……………………………?

b) Treatment protocol (Specific/Need based)

I) Name:

a)

b)

c)

d)

II) Structure (Injured ⟷ Rx):

a)

b)

c)

d)

III) Dose:

a)

b)

c)

d)

IV) Outcome

I) Patient’s perception: ____

II) Physiotherapist’s perception: ____

Day-4

a) General pain intensity ……………………………. ?

b) Treatment protocol (Specific/Need based)

I) Name:

a)

b)

c)

d)

II) Structure (Injured ⟺ Rx):

a)

b)

c)

d)

III) Dose:

a)

b)

c)

d)

IV) Outcome

I) Patient's perception: ____

II) Physiotherapist's perception: ______________________________________

Day-5

a) General pain intensity ……………………………. ?

b) Treatment protocol (Specific/Need based)

I) Name:

a)

b)

c)

d)

II) Structure (Injured ⟺ Rx):

a)

b)

c)

d)

III) Dose:

a)

b)

c)

d)

IV) Outcome

I) Patient’s perception: ____

II) Physiotherapist’s perception: ____
